# Mesenteroaxial gastric volvulus associated with a wandering spleen in an older woman treated with anterior gastropexy and splenectomy: a case report

**DOI:** 10.1007/s12328-026-02415-z

**Published:** 2026-08-07

**Authors:** Hirotaka Nomiya, Kazuya Ofuji, Takuto Nosaka, Arisa Tsuji, Ryotaro Sugata, Tadayoshi Otsuki, Seiichi Taguchi, Yoshiaki Imamura, Yasunari Nakamoto, Hiroyuki Suto

**Affiliations:** 1 Department of Gastroenterology, Japan Community Healthcare Organization Fukui Katsuyama General Hospital, 2-6-21 Nagayama-cho, Katsuyama, Fukui Japan; 2 Second Department of Internal Medicine, Faculty of Medical Sciences, University of Fukui, 23-3 Matsuoka Shimoaizuki, Eiheiji-cho, Yoshida-gun, Fukui Japan; 3 Department of Pathology, University of Fukui Hospital, 23-3 Matsuoka Shimoaizuki, Eiheiji-cho, Yoshida-gun, Fukui Japan

**Keywords:** Gastric volvulus, Wandering spleen, Splenic torsion, Gastropexy, Splenectomy

## Abstract

An 80-year-old woman with Parkinson disease presented with sudden abdominal pain, back pain, and vomiting during a meal. Computed tomography revealed marked gastric dilatation with cranial displacement and rotation of the antrum, consistent with mesenteroaxial gastric volvulus. This was accompanied by an ectopic spleen adjacent to the greater curvature and suspected pedicle torsion, consistent with a wandering spleen. Nasogastric tube passage was initially unsuccessful. Emergency upper gastrointestinal endoscopy achieved gastric decompression and transient detorsion; however, immediate retorsion was observed, and continuous decompression was maintained after successful tube placement. Definitive surgery was performed on hospital day 2. The stomach was reduced and fixed to the abdominal wall via anterior gastropexy; splenectomy was performed due to marked splenic mobility and concerns about recurrent torsion. Histopathological examination revealed congestion and partial hemorrhagic infarction compatible with splenic torsion. The postoperative course was uneventful; the patient was discharged on postoperative day 14 without recurrence during the follow-up. This case underscores that immediate retorsion after endoscopic reduction might warrant prompt definitive surgery with gastropexy and management of a wandering spleen should be individualized according to mobility and splenic viability.

## Introduction

Gastric volvulus is an uncommon but potentially life-threatening cause of acute abdomen. Abnormal rotation of the stomach can result in gastric outlet obstruction and, in severe cases, strangulation with ischemia or perforation [1]. The condition is commonly classified by the axis of rotation (organoaxial, mesenteroaxial, or combined) and also categorized as primary (idiopathic) or secondary to underlying anatomic abnormalities that alter normal gastric fixation. In acute cases, clinical deterioration can be rapid; therefore, early recognition and timely intervention remain essential [1].

The clinical manifestations of gastric volvulus ranges between intermittent postprandial symptoms to acute abdomen. Although Borchardt triad, comprising severe epigastric pain with distention, unproductive retching, and inability to pass a nasogastric tube, is a classic diagnostic clue, it might be incomplete [2]. Initial management generally prioritizes decompression and reduction to relieve obstruction and mitigate ischemic risk. Endoscopic decompression with attempted reduction may serve as a temporizing approach in selected patients; however, recurrence after conservative measures is common, and definitive surgical treatment is frequently required for preventing recurrent volvulus [1, 2].

Wandering spleen is a rare condition characterized by ectopic splenic location due to abnormal mobility arising from laxity, elongation, or absence of the splenic suspensory ligaments, predisposing to torsion with vascular compromise and infarction [3, 4]. The coexistence of gastric volvulus and wandering spleen has been described and may reflect shared abnormalities of visceral attachments, with a mobile spleen potentially exerting traction on the stomach, particularly along the greater curvature [5–7]. Adult presentations appear uncommon compared with pediatric reports [3, 4]. Herein, we describe an older woman with suspected mesenteroaxial gastric volvulus associated with a wandering spleen.

## Case report

An 80-year-old woman with Parkinson disease and bronchial asthma presented to our emergency department with acute abdominal and back pain during a meal, accompanied by vomiting. She had no history of abdominal surgery. Her regular medications included levodopa/carbidopa (600 mg/day), opicapone (25 mg/day), and safinamide (100 mg/day). The patient was receiving inhaled fluticasone furoate/vilanterol (Relvar Ellipta 200) for bronchial asthma, without asthma attacks in recent years. The patient had no known drug allergies; she did not smoke; however, she occasionally consumed alcohol.

On arrival, the patient showed height, 148.0 cm and weight, 46.4 kg (body mass index 21.2 kg/m^2^). The patient exhibited body temperature, 36.5 °C; blood pressure, 140/90 mmHg; pulse, 96 beats/min; and oxygen saturation, measured by pulse oximetry, 95% on room air. She was alert, without conjunctival pallor or scleral icterus. On auscultation, the heart sounds were regular without murmurs, breath sounds were clear; there was no costovertebral angle tenderness. The abdomen was markedly distended and tympanitic, with diffuse tenderness and rigidity.

**Fig. 1:**
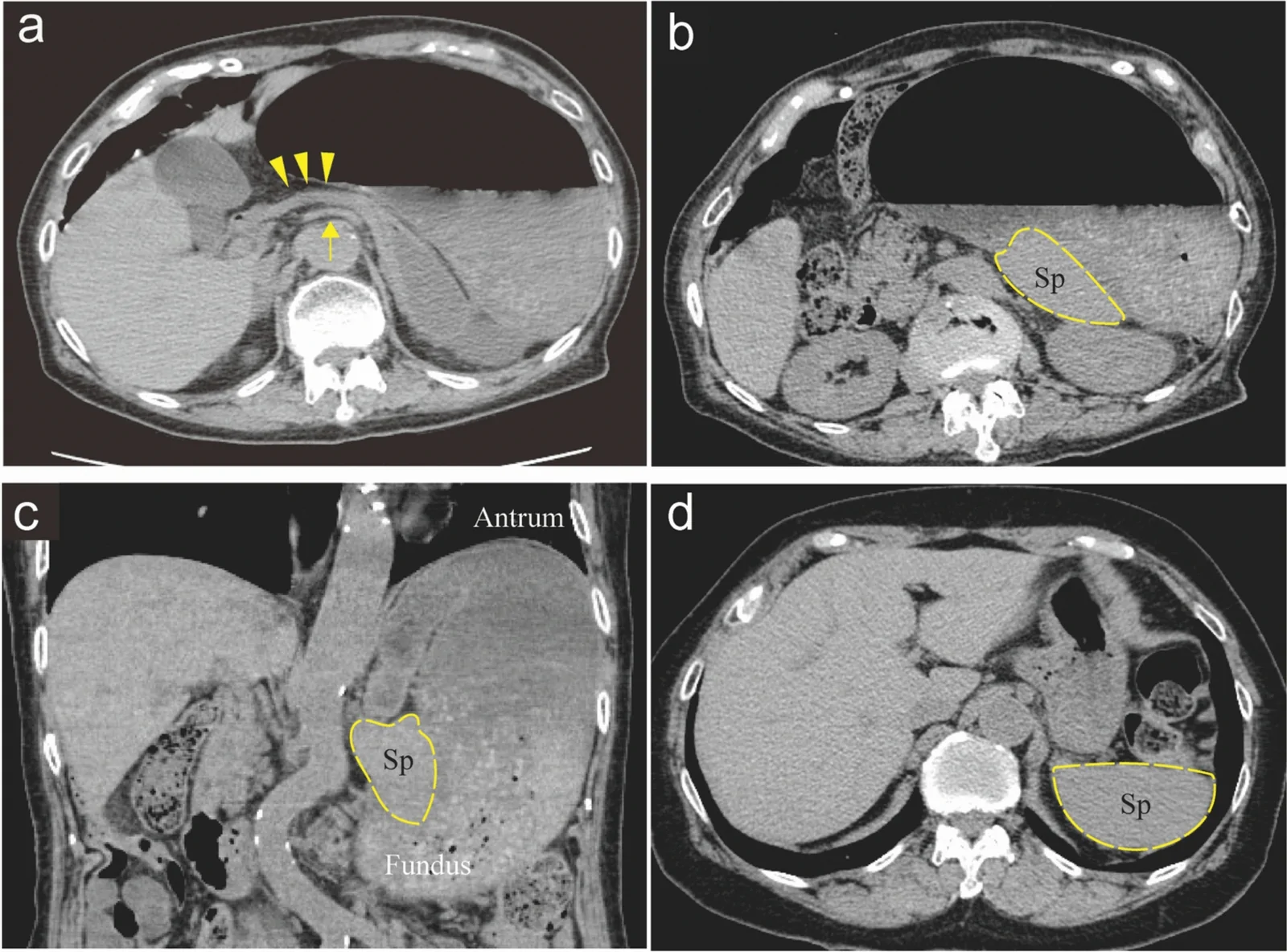
Computed tomography findings. **a** Axial computed tomography (CT) reveals marked gastric dilatation with duodenal distension (arrowheads) and narrowing of the esophagus (arrow). **b** Axial CT shows an ectopic spleen (Sp) outlined in yellow adjacent to the greater curvature, consistent with a wandering spleen. **c** Coronal CT demonstrates cranial displacement of the antrum above the fundus with an ectopic spleen (Sp) outlined in yellow, consistent with mesenteroaxial gastric volvulus. **d** Axial CT obtained 3 years prior to presentation shows the spleen (Sp) in its normal position within the left upper quadrant, suggesting an acquired increase in splenic mobility over time

Laboratory test results on admission revealed no major abnormalities (Table 1). These included white blood cell count, 9100/µL; hemoglobin, 13.2 g/dL; platelet count, 253 × 10^3^/µL; and C-reactive protein, 0.02 mg/dL. Contrast-enhanced computed tomography (CT) was not performed due to the patient’s history of bronchial asthma. Unenhanced CT revealed marked gastric dilatation with duodenal distension and esophageal narrowing (Fig. 1a). Coronal reconstruction demonstrated cranial displacement and rotation of the antrum; the duodenum passed anterior to the esophagus, indicating mesenteroaxial gastric volvulus (Fig. 1c). The spleen was ectopically located adjacent to the greater curvature with suspected pedicle torsion, consistent with a wandering spleen complicated by splenic torsion (Fig. 1b, c). Review of an abdominal CT performed 3 years earlier showed the spleen in a normal position in the left upper quadrant (Fig. 1d). Based on these imaging findings, mesenteroaxial gastric volvulus associated with a wandering spleen complicated by splenic torsion was diagnosed.

**Table 1:** Laboratory test results

Peripheral blood			Biochemistry		
WBC	9100 /μL		Na	141 mmol/L	
RBC	4.08 × 10^6^ /μL		K	3.4 mmol/L	
Hb	13.2 g/dL		Cl	101 mmol/L	
Plt	253 × 10^3^ /μL		Ca	9.0 mg/dL	
*Coagulation*			Total protein	7.4 g/dL	
PT	10.9 s		Albumin	4.2 g/dL	
PT-INR	1.04		UN	17.1 mg/dL	
APTT	23.6 s		Creatinine	0.64 mg/dL	
*Viral markers*			Total bilirubin	1.3 mg/dL	
HBs-Ag	<0.05 IU/mL		Direct bilirubin	0.6 mg/dL	
HBs-Ab	<10.00 mIU/mL		AST	14 U/L	
HBc-Ab	7.72 C.O.I		ALT	4 U/L	
HCV-Ab	(–)		LDH	179 U/L	
			ALP	50 U/L	
			γ-GTP	13 U/L	
			Amylase	86 U/L	
			CRP	0.02 mg/dL	

Blood test results at the time of admission

WBC, white blood cell; RBC, red blood cell; Hb, hemoglobin; Plt, platelet; PT, prothrombin time; PT-INR, prothrombin time-international normalized ratio; APTT, activated partial thromboplastin time; HBs-Ag, hepatitis B surface antigen; HBs-Ab, hepatitis B surface antibody; HBc-Ab, hepatitis B core antibody; HCV-Ab, hepatitis C virus antibody; UN, urea nitrogen; AST, aspartate aminotransferase; ALT, alanine aminotransferase; LDH, lactate dehydrogenase; ALP, alkaline phosphatase; γ-GTP, glutamyl transpeptidase; CRP, C-reactive protein

**Fig. 2:**
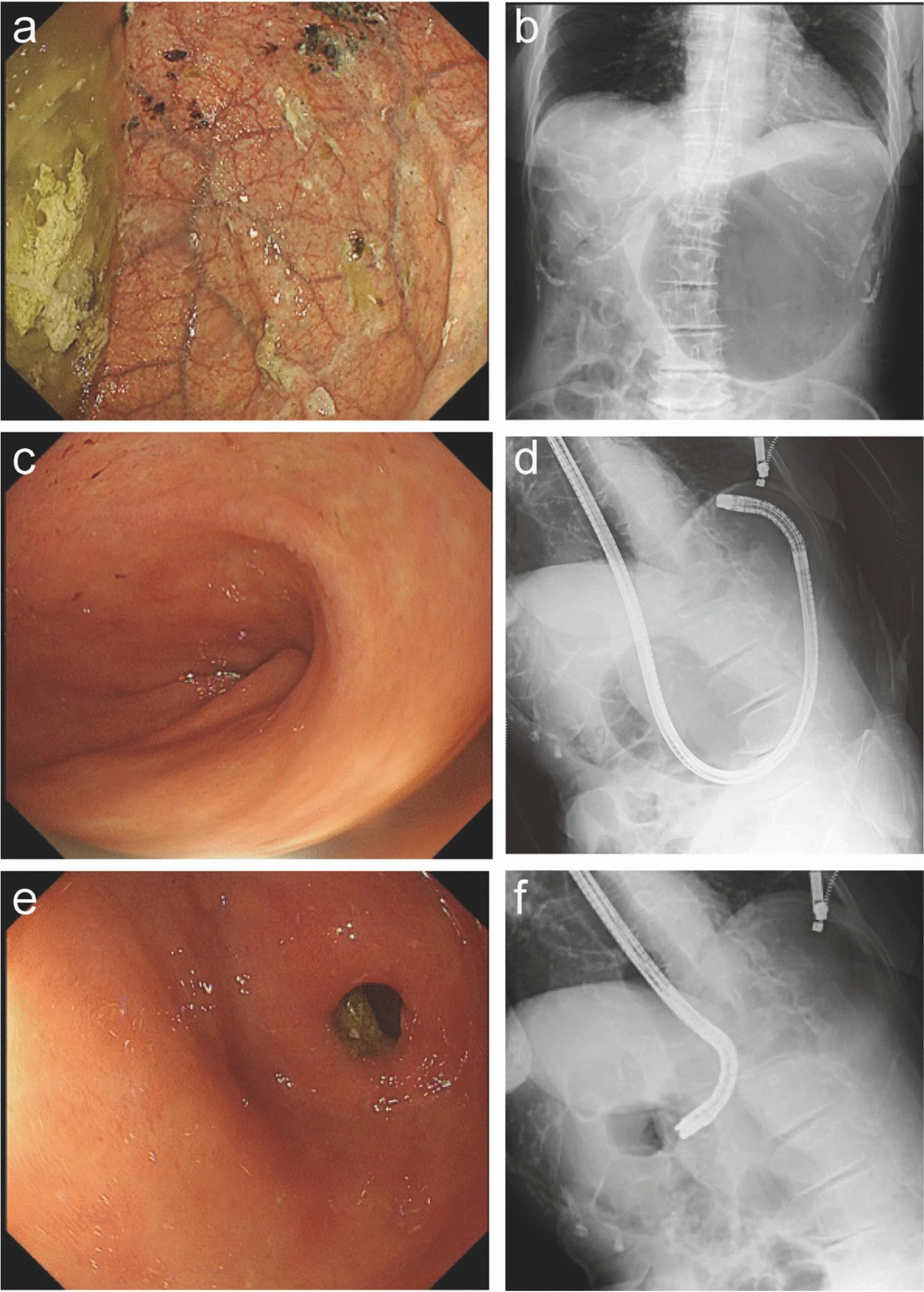
Upper gastrointestinal endoscopy, radiography, and fluoroscopic findings on hospital day 1. **a** Endoscopic view of the gastric mucosa without obvious ischemia. **b** Plain abdominal radiograph demonstrates marked gastric dilatation. **c** Endoscopic view shows torsion-related luminal distortion. **d** Fluoroscopy during endoscopy shows the endoscope advanced to the pylorus with a pushing maneuver. **f** Fluoroscopy after detorsion shows transient normalization of the gastric configuration. **e** Endoscopic view of the pylorus after detorsion

Passage of a nasogastric tube was attempted but was unsuccessful due to strong resistance. Emergency upper gastrointestinal endoscopy was performed for gastric decompression and attempted reduction. The gastric lumen was markedly dilated; torsion-related luminal distortion was observed. However, endoscopic assessment did not reveal obvious mucosal ischemia (Fig. 2a–c). The endoscope was advanced to the duodenum with a pushing maneuver (Fig. 2c, d); detorsion was achieved by rotating the scope in the direction opposite to that of the torsion (Fig. 2e, f); however, immediate retorsion was observed. Following endoscopic decompression, a gastric tube was successfully placed and continuous decompression was maintained, resulting in symptom relief. Given the immediate retorsion after endoscopic reduction and suspected splenic torsion, definitive surgical treatment was planned.

**Fig. 3:**
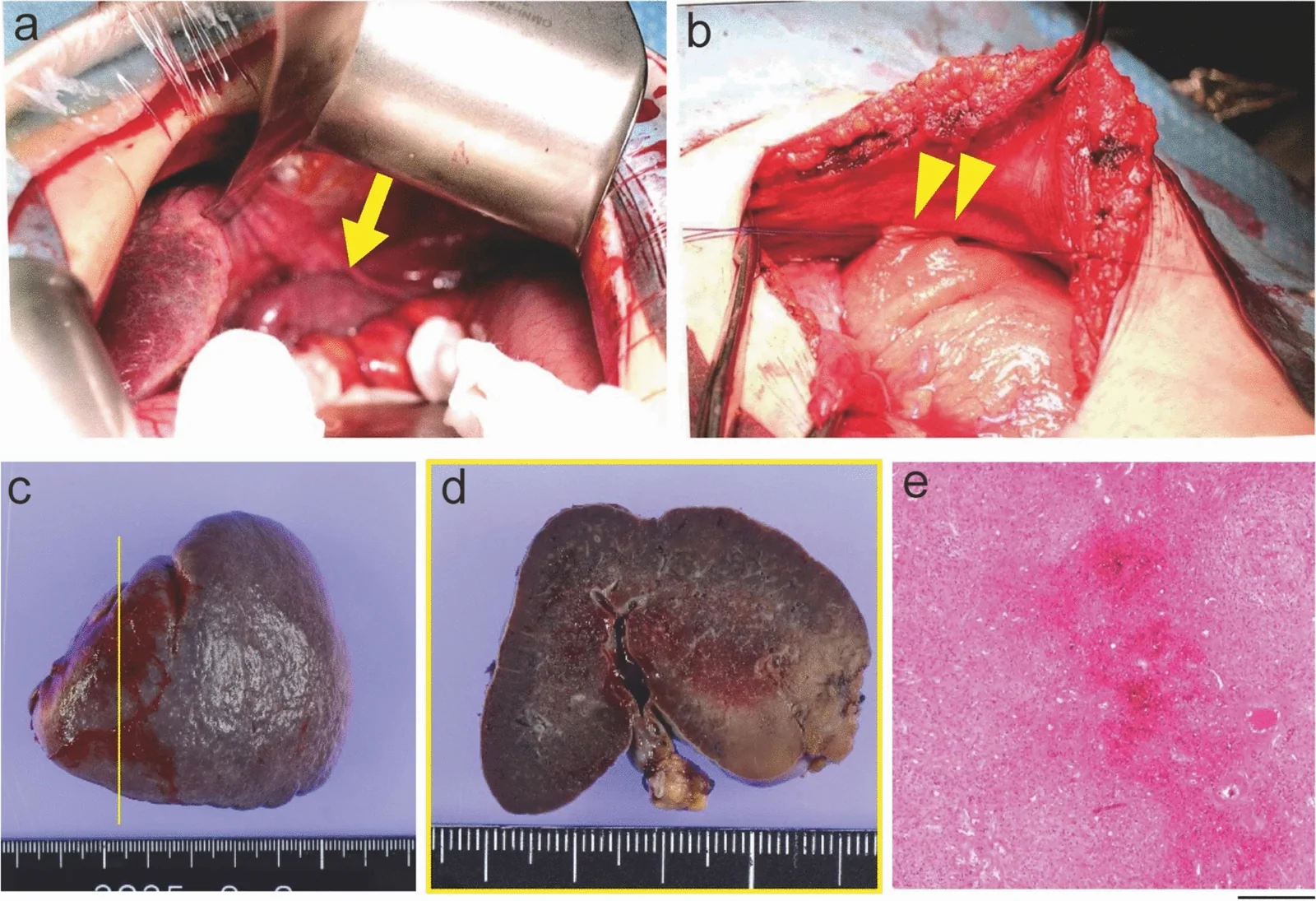
Intraoperative, macroscopic, and histopathological findings. **a** Intraoperative view shows the ectopic spleen located behind the stomach (arrow). **b** Anterior gastropexy is performed by fixing the anterior gastric wall to the abdominal wall (arrowheads). **c** Macroscopic appearance of the resected spleen shows marked congestion; the yellow line indicates the section plane. **d** Cut surface shows areas of hemorrhage and partial hemorrhagic infarction. **e** Hematoxylin and eosin staining shows red pulp congestion and hemorrhage. H&E, hematoxylin and eosin

On hospital day 2, open surgery was performed. The spleen was markedly mobile and located behind the stomach, suggesting traction-related contribution to the volvulus (Fig. 3a). The gastric volvulus was reduced; furthermore, the anterior gastric wall was fixed to the abdominal wall (anterior gastropexy) to prevent recurrence (Fig. 3b). Splenectomy was performed because fixation was considered technically unsafe given the degree of mobility and concerns about recurrent torsion. Histopathological examination of the resected spleen demonstrated red pulp enlargement with prominent congestion, hemorrhage, and partial hemorrhagic infarction, compatible with splenic torsion (Fig. 3c–e). The postoperative course was uneventful; the patient was discharged on postoperative day 14. Because the splenectomy was performed as a semi-emergency procedure, preoperative vaccination against encapsulated organisms was not administered. No recurrence or infection was observed during the 12 months follow-up.

## Discussion

The coexistence of gastric volvulus and wandering spleen has been reported predominantly in pediatric cases [3, 4]; however, adult presentations remain rare, with only isolated cases and small case series reported [5–9]. Previously reported adult cases with available individual clinical information are summarized in Table 2 [5, 8, 10–15]. A review of these reported cases demonstrated that very elderly patients were uncommon; the present case is among the oldest reported. All cases were of the mesenteroaxial type; severe complications such as gastric ischemia or necrosis were frequently observed, and nearly all cases required surgical intervention. Recognition of this association is clinically relevant because therapeutic strategy should address not only the malrotated stomach, but also an underlying highly mobile spleen that might contribute to repeated torsion through traction along the greater curvature [6, 7]. In our case, unenhanced CT revealed both the configuration of the stomach and ectopic spleen with suspected torsion, enabling etiologic diagnosis at presentation. In addition, review of a CT scan obtained 3 years earlier demonstrated a normally positioned spleen, suggesting an acquired increase in splenic mobility over time. The splenorenal (lienorenal) ligament forms a dorsal mesenteric attachment between the splenic hilum and posterior abdominal wall, through which the distal splenic vessels and pancreatic tail pass [16]. Pancreatic volume decreases with age, and pancreatic atrophy becomes more apparent after the age of 60, particularly in the tail [17–19]. Therefore, age-related atrophy of the pancreatic tail might reduce support around the splenic hilum and contribute to increased splenic mobility in susceptible individuals; however, no previous report has directly demonstrated this mechanism, and this remains a speculative possibility.

**Table 2:** Previously reported adult cases of gastric volvulus associated with a wandering spleen

References	Age/sex	Background	Borchardt’s Triad (P/V/NG)	Episodes	Type	Initial management	Definitive treatment	Complications	Outcome
Lianos et al. [10]	28/F	None	+ / + / +	Initial	Mesenteroaxial	NG, EGD	Open gastropexy	Gastric ischemia	No recurrence, 2 months
Ooka et al. [11]	22/M	Wilson disease	+ /NR/NR	Recurrent	Mesenteroaxial	NG	No definitive fixation	None	No recurrence, 6 months
Tada et al. [12]	58/M	Poliomyelitis	+ / + /NR	Initial	Mesenteroaxial	EGD	Total gastrectomy	Gastric necrosis	Survived
Omata et al. [5]	43/F	None	+ / + / +	Recurrent	Mesenteroaxial	NG, endoscopic reduction	Laparoscopic phrenofundopexy and anterior gastropexy	None	No recurrence, 24 months
Hamada et al. [13]	66/F	None	+ / + /NR	Initial	Mesenteroaxial	NG, endoscopic reduction	Open gastropexy and omental covering	Gastric ulcer perforation	No recurrence
Basu et al. [8]	65/F	None	+ / + / +	Recurrent	Mesenteroaxial	NG	Segmental gastric resection, pyloroplasty, and splenectomy	Gastric necrosis	No recurrence, 3 months
Belayneh et al. [14]	53/F	None	+ / + /NR	Recurrent	Mesenteroaxial	NR	Open proximal subtotal gastrectomy, esophagogastric anastomosis, and splenopexy	Gastric perforation and necrosis	No recurrence, 1 month
Slaoui et al. [15]	58/M	None	+ / + /NR	Initial	Mesenteroaxial	NR	Open gastropexy and splenopexy	NR	No recurrence, 3 months
Present case	80/F	Parkinson disease	+ / + / +	Initial	Mesenteroaxial	NG, endoscopic reduction	Open anterior gastropexy and splenectomy	Splenic hemorrhagic infarction	No recurrence, 12 months

P/V/NG indicates the three components of Borchardt’s triad: abdominal pain or abdominal distension, vomiting or retching, and difficulty or inability to pass a nasogastric tube, respectively. “ + ” indicates that the component was reported. NR indicates not reported. EGD, esophagogastroduodenoscopy; NG, nasogastric tube. Initial management denotes decompression or conservative management before definitive surgery. Outcomes are shown as reported in each article

**Fig. 4:**
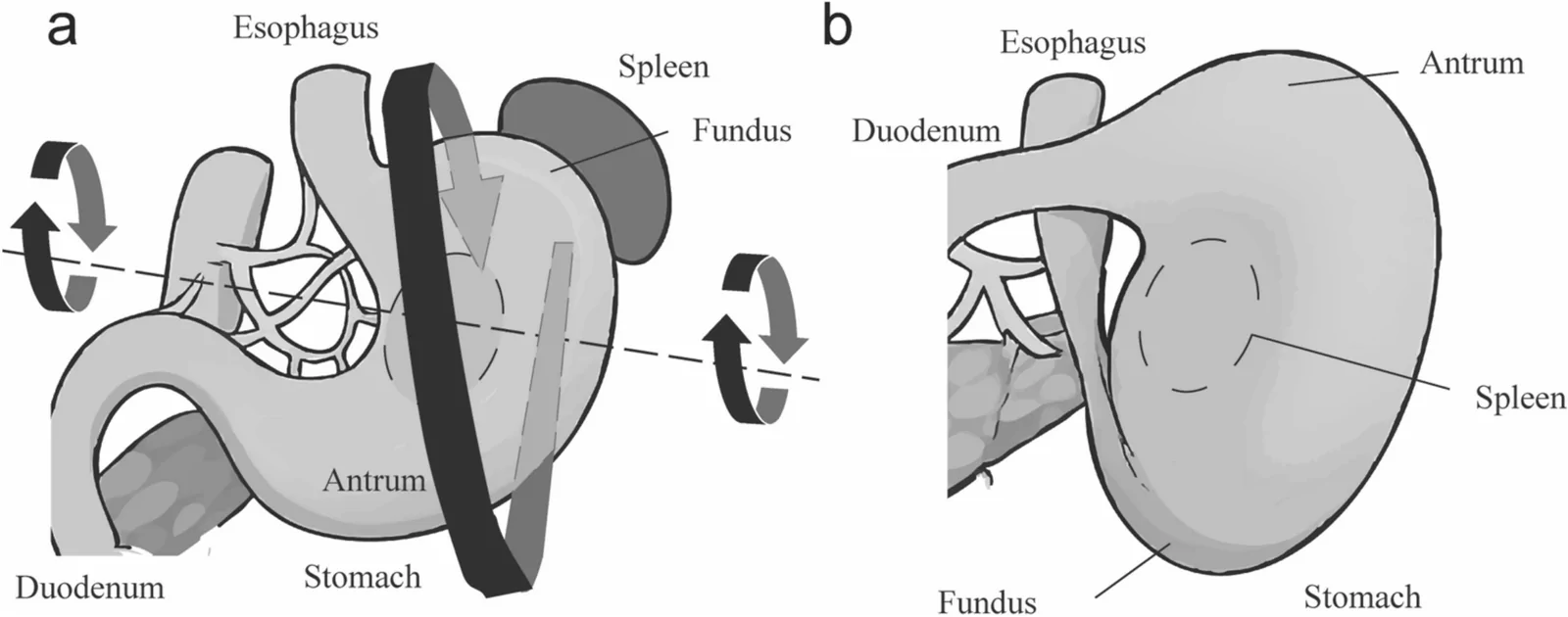
Schematic illustrations of mesenteroaxial gastric volvulus associated with a wandering spleen. **a** Schematic illustration shows the normal anatomical relationship between the stomach and spleen. The dashed line indicates the axis of mesenteroaxial rotation, and the arrows indicate the direction of gastric rotation. The highly mobile spleen may move along the greater curvature in the same direction as the stomach, thereby exerting traction that promotes mesenteroaxial volvulus. **b** Schematic illustration shows the relationship between the malrotated stomach and ectopic spleen

Gastric volvulus is most commonly the organoaxial type, in which the stomach rotates around its long axis, whereas the less common mesenteroaxial type involves rotation around the short axis with anterior and superior displacement of the antrum and pylorus [1, 2]. In our case, CT demonstrated the antrum cranially displaced above the fundus with the duodenum passing anterior to the esophagus, consistent with mesenteroaxial rotation (Fig. 1c). Notably, several reported cases of gastric volvulus associated with wandering spleen have involved the mesenteroaxial type [5–7, 9], possibly because splenic traction along the greater curvature might preferentially promote rotation around the short axis. Based on CT and intraoperative findings, the wandering spleen was presumed to have moved around the anterior aspect of the stomach along the greater curvature and then shifted posterior to the stomach (Fig. 4). This movement may have exerted traction on the greater curvature and promoted mesenteroaxial gastric volvulus, while the final posterior position of the spleen may have contributed to splenic pedicle torsion and early retorsion after endoscopic reduction. Because this mechanism was inferred from CT and intraoperative findings, it should be regarded as a schematic hypothesis rather than a directly observed dynamic process. In patients with acute gastric volvulus, early diagnosis is important because delayed management could be associated with ischemia, necrosis, and perforation [1]. In our case, endoscopy was valuable for decompressing the stomach and evaluating mucosal viability; however, the immediate retorsion indicated persistence of the underlying mechanical predisposition.

Endoscopic decompression and attempted reduction can serve as an initial measure, particularly to stabilize patients and bridge to definitive surgery [1, 5]. In a series of 44 patients with chronic gastric volvulus managed conservatively without anatomical correction, symptomatic recurrence occurred in 28 patients (64%) during a median follow-up of 16 months [20]. Therefore, endoscopic reduction should be regarded as a temporizing intervention rather than a definitive treatment when an anatomical predisposing factor, such as a wandering spleen, remains uncorrected. In our case, definitive surgery was considered appropriate based on three factors: immediate retorsion after transient endoscopic reduction, radiologic evidence of a wandering spleen with suspected torsion, and the need for a durable strategy for preventing recurrence. Anterior gastropexy was performed to limit gastric mobility and prevent retorsion, consistent with established surgical principles in gastric volvulus [1]. Although minimally invasive approaches have been reported [5], open surgery was selected for timely definitive management in the setting of recurrent torsion and suspected concomitant splenic torsion. Management of wandering spleen is guided by the risk of torsion and splenic viability. Splenopexy is generally favored when the spleen is viable to preserve splenic function, whereas splenectomy is reserved for infarcted or nonviable spleens or when fixation is not feasible or is considered unsafe [3, 4]. Although the risk of post-splenectomy infection was an important concern, splenectomy was selected because the spleen was markedly mobile, and splenic torsion with ischemia was suspected. Moreover, pathology demonstrated congestion and partial hemorrhagic infarction consistent with torsion.

This report has limitations. Contrast-enhanced CT was not performed; although bronchial asthma alone is not a contraindication to iodinated contrast media, we deferred it because unenhanced CT had already established the diagnosis and endoscopy had confirmed the absence of gastric ischemia. Nonetheless, this case highlights that gastric volvulus associated with a wandering spleen could occur in very elderly patients and might demonstrate immediate retorsion after endoscopic reduction. These findings suggested that confirming the splenic position on CT was important even in older patients presenting with gastric volvulus, considering the possibility of a wandering spleen. Awareness about this association is important for timely diagnosis and selecting definitive management that addresses both gastric fixation and splenic mobility.
